# Acute Exercise on Memory Reconsolidation

**DOI:** 10.3390/jcm8081200

**Published:** 2019-08-11

**Authors:** Paul D. Loprinzi, Ashley Lovorn, Emma Hamilton, Noelle Mincarelli

**Affiliations:** Exercise & Memory Laboratory, Department of Health, Exercise Science and Recreation Management, The University of Mississippi, Oxford, MS 38677, USA

**Keywords:** episodic memory, intrusions, memory distortion

## Abstract

Background and Objective: Once a memory is reactivated, it enters a labile state and, thus, is vulnerable to memory decay and/or distortion. Recent research demonstrates that acute, high-intensity exercise is associated with enhanced episodic memory function. Very limited research, however, has evaluated whether acute exercise can attenuate memory distortion from memory reactivation, which was the purpose of this study. Methods: A between-subject randomized controlled intervention was employed. Participants (N = 80) were randomly assigned to one of four groups, including (1) reminder with exercise, (2) reminder, (3) no reminder, and (4) interference control. For the groups, participants completed three visits (Visit 1, 2, and 3), which all occurred 48 hours apart. An exception to this was the interference control group, which did not complete Visit 2. On Visit 2, the reminder with exercise group engaged in a 15 min bout of high-intensity exercise (80% of heart rate reserve) immediately after memory reactivation. On Visit 3, participants engaged in a free recall (4 trials) of the memory task encoded on Visit 1. Results: In a 4 (groups) × 4 (learning trials) mixed-measures ANOVA, with the group as the between-subjects variable and the learning trials (1–4) as the within-subject variable, there was a significant main effect group, *F*(3, 76) = 4.18, *p* = 0.008, η^2^_p_ = 0.14, and a significant main effect for the learning trials, *F*(2.40, 182.59) = 49.25, *p* < 0.001, η^2^_p_ = 0.39, but there was no group by learning trials interaction, *F*(7.20, 182.59) = 1.07, *p* = 0.38, η^2^_p_ = 0.04. Conclusion: Our findings suggest that exercise may, potentially, attenuate memory distortion from memory reactivation. However, future work is needed to confirm these findings before any strong conclusions can be reached.

## 1. Introduction

In a recent meta-analysis [1], along with several experimental studies [2,3,4], we showed that acute exercise, particularly high-intensity exercise [5,6,7], is effective in enhancing episodic memory function (retrospective recall of information). We have previously discussed the potential mechanisms of this effect [8,9], which likely results from exercise-induced increases in neuronal excitability [10], as well as production of key proteins (e.g., brain-derived neurotrophic factor) [11,12,13,14].

The timing of the acute bout of exercise appears to have an important effect on episodic memory function. Evidence suggests that when the acute bout of exercise occurs during memory encoding, memory function may be impaired [1]. However, if the bout of exercise occurs shortly before memory encoding or during memory consolidation, long-term memory function may be enhanced [1]. Notably, research demonstrates that if an acute bout of exercise occurs either during the early stages of memory consolidation (e.g., immediately after encoding) [1] or during the later stages of consolidation (e.g., 4 h post-encoding) [1,15], then long-term memory may be enhanced.

Per the memory reconsolidation theory [16], once a memory is reactivated, it enters a labile state and, thus, is vulnerable to memory decay and/or distortion. Although exercise during the memory consolidation stage has been shown to enhance memory retention [1,15], we have little experimentation as to whether exercise can attenuate memory distortion from memory reactivation, which was the purpose of this study. We hypothesized that after memory reactivation, engaging in an acute bout of high-intensity exercise would result in greater delayed memory recall, when compared to memory reactivation without exercise. This effect is plausible, as both memory consolidation and memory reconsolidation require protein synthesis after learning and reactivation [17]. Acute high-intensity exercise may assist in memory reconsolidation given its established effect in facilitating neuronal protein synthesis in the hippocampus [18].

## 2. Methods

### 2.1. Study Design

A between-subject randomized controlled intervention was employed. Participants were randomly assigned to one of four groups. Protocol details of these groups are discussed in the Study Procedures section (below). This study was ethically approved by the authors’ institutional review board. Participants provided written consent prior to participation.

### 2.2. Participants

The study included 80 participants (n = 20 per group), which is approximately twice the per group size of other related reconsolidation studies [19]. Recruitment occurred via a convenience-based, nonprobability sampling approach (classroom announcement and word-of-mouth). Participants included undergraduate and graduate students between the ages of 18 and 40 years.

Additionally, participants were excluded if they:
Self-reported as a daily smoker [20,21];
Self-reported being pregnant [22];
Exercised within 5 h of testing [23];
Consumed caffeine within 3 h of testing [24];
Had a concussion or head trauma within the past 30 days [25];
Took marijuana or other illegal drugs within the past 30 days [26];
Were considered a daily alcohol user (>30 drinks/month for women; >60 drinks/month for men) [27].

### 2.3. Exercise Assessment

The exercise bout included a 15 min treadmill session at 80% of heart rate reserve. This constitutes vigorous exercise intensity [28]. Heart rate was measured throughout the exercise bout. Treadmill speed/incline were manipulated to ensure the participant’s heart rate stayed within 5 beats per minute of the target heart rate.

### 2.4. Memory Assessment

Table 1 displays the list of objects presented on Visit 1 and Visit 2. Further details are discussed in the Protocol for Visits section (below).

### 2.5. Protocol for Visits

Participants were randomly assigned to one of four groups, including (1) reminder with exercise, (2) reminder, (3) no reminder, and (4) interference control. For the groups, participants completed three visits (Visit 1, 2, 3), which all occurred 48 hours apart from each other. An exception to this was the interference control group, which did not complete Visit 2. See Table 2 below for a schematic of the protocol, which is fully detailed in the narrative that follows.

VISIT 1 PROTOCOL:

The protocol for all four groups was identical for Visit 1.

On Visit 1, the researcher pulled out one item at a time from a bag and placed it in a distinctive blue basket (items are from List 1). Participants were asked to name each item and to pay close attention so they could remember the items later. After 20 items were placed into the basket, the researcher hid the basket and asked the participants to try and recall as many items as possible. This protocol was repeated until the participant remembered at least 17 of the items or until a maximum of four learning trials occurred. The number of learning trials was recorded.

VISIT 2 PROTOCOL:

The procedure on Visit 2 differed across the groups.

**Reminder Group**: For those in the reminder group, on Visit 2, the same researcher who administered the protocol on Visit 1 showed them the empty blue basket and asked, “*Do you remember this basket and what we did with it?*” Participants were encouraged to describe the procedure but were stopped if they started to recall any specific items.

They then learned a second list of objects (List 2). The procedure differed from List 1, so the task did not serve as a reminder. All objects (List 2) were placed in front of the participant, and the participant was asked to name each of the objects and were given 30 sec to study the objects before naming each object. The researcher then had the participants face away from the objects (so they could not see them) and asked the participant to recall as many of the objects as possible. If the participant recalled <17 objects, they were re-exposed to the objects for another 30 sec. This repeated until the participant recalled at least 17 objects or for a maximum of four learning trials. The number of learning trials was recorded.

**Reminder Group Plus Exercise**: Those in the reminder group plus exercise completed the same procedure as the reminder group, but then after the memory task, they engaged in a 15 min bout of treadmill exercise at 80% of heart rate reserve. Thus, the reminder group and the reminder plus exercise group were identical, with the exception that the reminder plus exercise group engaged in a bout of exercise after learning List 2. The idea here is that the bout of exercise would help to attenuate an intrusion effect.

**No Reminder Group**: For those in the no reminder group, on Visit 2, a different researcher administered the experimental procedure in a different room. The researcher did not ask what had happened on Visit 1 nor presented the blue basket.

They then learned a second list of objects (List 2). The procedure differed from List 1, so the task did not serve as a reminder. All objects (List 2) were placed in front of the participant, and the participant was asked to name each of the objects and were given 30 sec to study the objects before naming each object. The researcher then had the participant face away from the objects and asked the participant to recall as many of the objects as possible. If the participant recalled <17 objects, the participant was re-exposed to the objects for another 30 sec. This was repeated until the participant recalled at least 17 objects or for a maximum of four learning trials. The number of learning trials was recorded.

**Interference Control**: Those in the interference control group did not complete a Visit 2 visit.

VISIT 3 PROTOCOL:

The protocol for all four groups was the same for Visit 3.

The researcher who administered the protocol on Visit 1 completed the protocol on Visit 3. This researcher asked the participant to recall as many objects as possible from Visit 1. When the participant indicated that they could not recall any more objects, they completed simple arithmetic problems for 30 sec. After this 30-sec distraction period, the researcher had the participant repeat the recall test by asking them to recall the objects again. This procedure was repeated for a total of four consecutive trials to test the reliability of the recall.

For the Visit 3 assessment, the outcome measures included the words recalled from List 1 as well as the number of intrusions, i.e., the number of items that were falsely recalled from List 2 (i.e., the number of items they stated from List 2).

### 2.6. Statistical Analysis

All statistical analyses were computed in JASP (v. 0.9.2.0). Descriptive statistics are reported as arithmetic means and their corresponding standard deviations. A 4 (condition) × 4 (trials) two-factor mixed-measures ANOVA was computed for the List 1 recall on Visit 3. A 3 (condition) × 4 (trials) two-factor mixed-measures ANOVA was computed for the List 2 intrusions on Visit 3. Lastly, a one-factor repeated-measures ANOVA was computed for the heart rate response from exercise. In all ANOVA models, the sphericity assumption was violated, and as such, for all ANOVA models, we report the Huynh–Feldt corrected values. Statistical significance was set at an alpha of 0.05. Partial-eta squared (η^2^_p_) was calculated as an effect size estimate for the ANOVA models, whereas Cohen’s d was calculated as an effect size estimate for the post-hoc analyses.

## 3. Results

### 3.1. Demographic Characteristics

Table 3 displays the demographic characteristics of the sample. Participants, on average, were 20.5 (2.4) years of age and had an average BMI of 24.9 (4.1) kg/m^2^. The sample was predominately female (67.5%) and non-Hispanic white (88.8%). Notably, there were no statistically significant differences in these demographic parameters across the sample.

An ANOVA was used to calculate the *p*-value for continuous variables (e.g., age), whereas a chi-square test was used to calculate the *p*-value for categorical variables (e.g., gender).

#### 3.1.1. Performance on Visit 1: Acquisition of List 1

Here we report the number of learning trials that were necessary for participants to recall at least 17 objects. Participants who recalled <17 objects during the fourth learning trial were given a score of 5. Participants, on average, took 2.54 (0.85) trials to reach this criterion. The mean number of trials across the reminder with exercise, reminder, no reminder, and interference control groups, respectively, was 2.70 (0.97), 2.47 (0.84), 2.42 (0.75), and 2.55 (0.88). There were no significant differences across groups, *F*(3, 76) = 0.38, *p* = 0.76, η^2^_p_ = 0.01.

#### 3.1.2. Performance on Visit 2: Acquisition of List 2

The mean number of trials across the reminder with exercise, reminder, and no reminder groups, respectively, was 2.65 (0.74), 2.42 (0.90), and 2.71 (0.56). There were no significant differences across groups, *F*(2, 57) = 0.84, *p* = 0.44, η^2^_p_ = 0.02.

#### 3.1.3. Exercise Response on Visit 2

**Figure 1:**
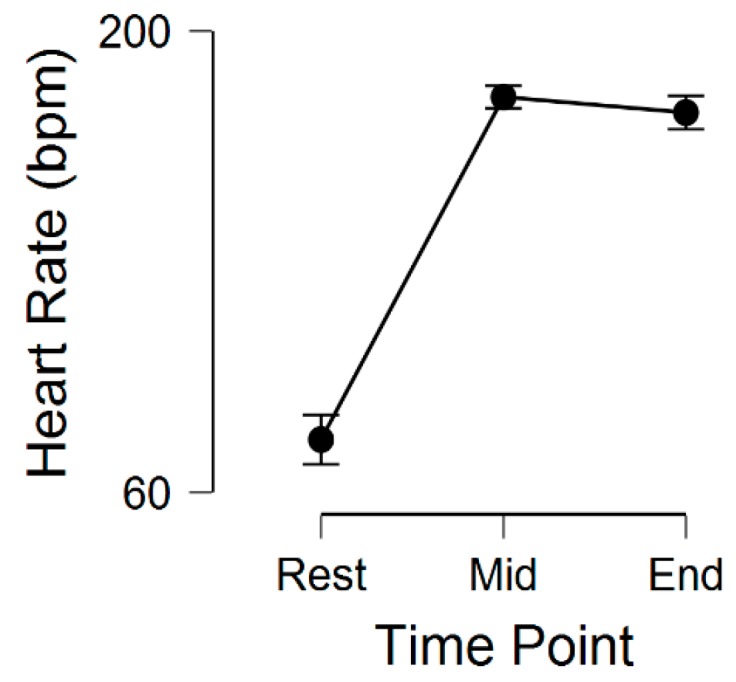
Physiological (heart rate) response to the acute bout (15 min) of treadmill exercise in the Reminder with Exercise group. Error bars represent 95% CI.

After the retrieval of List 2, the reminder with exercise group engaged in a 15 min bout of treadmill exercise at 80% of their heart rate reserve. A one-factor repeated-measures ANOVA demonstrated a significant main effect (for heart rate) across the three time-points (rest, midpoint of exercise, and immediately prior to the end of exercise), *F*(1.20, 22.87) = 485.3, *p* < 0.001, η^2^_p_ = 0.02. The mean heart rate across these three time-points was 76.0 (12.4), 179.9 (18.1), and 175.2 (20.9), respectively (Figure 1).

#### 3.1.4. Performance on Visit 3: Recall

The Cronbach’s alpha for the performance of the four learning trials for List 1 and intrusions from list 2, respectively, are, α = 0.97 and α = 0.98. The mean number of words recalled for the four List 1 trials was 8.20 (3.2), 8.91 (3.3), 9.63 (3.2), and 10.21 (3.3), respectively. The mean number of intrusions (words) for the four trials was 3.75 (4.6), 3.70 (4.7), 3.75 (5.0), and 3.78 (5.0), respectively.

The average number of List 1 words recalled (averaged across the four List 1 recall trials) for the reminder with exercise, reminder, no reminder, and interference control groups, respectively, was 9.09 (3.5), 8.51 (3.2), 8.17 (2.6), and 11.20 (2.5). The average number of intrusions (averaged across the four trials) for the reminder with exercise, reminder, and no reminder groups, respectively, was 3.70 (5.8), 4.52 (4.8), and 3.19 (4.2).

**Figure 2:**
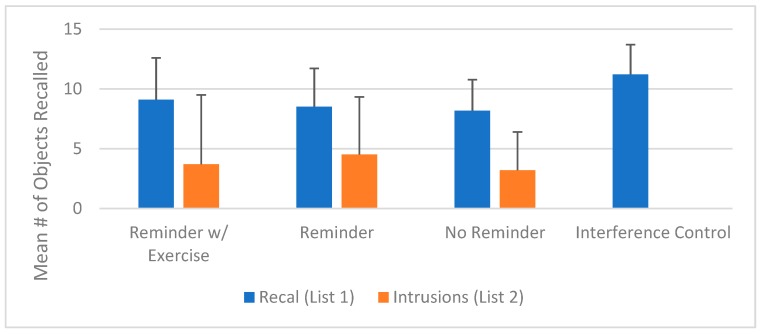
Mean number of objects recalled (List 1 and List 2) across the experimental conditions. Error bars represent standard deviations.

The mean number of items recalled from List 1 (averaged across the four recall trials) and the mean number of items falsely recalled from List 2 (intrusions, averaged across the four recall trials) is displayed in Figure 2.

#### 3.1.5. List 1 Recall

In a 4 (groups) × 4 (learning trials) mixed-measures ANOVA, with the group as the between-subjects variable and the learning trials (1–4) as the within-subject variable, there was a significant main effect for group, *F*(3, 76) = 4.18, *p* = 0.008, η^2^_p_ = 0.14, and a significant main effect for the learning trials, *F*(2.40, 182.59) = 49.25, *p* < 0.001, η^2^_p_ = 0.39, but there was no group by learning trials interaction, *F*(7.20, 182.59) = 1.07, *p* = 0.38, η^2^_p_ = 0.04.

Bonferroni-corrected post-hoc tests indicated that for the within-subject factor, trial 1 was different than trial 2 (*p* < 0.001, d = 0.45), trial 3 (*p* < 0.001, d = 0.83), and trial 4 (*p* < 0.001, d = 1.01). Similarly, trial 2 was different than trial 3 (*p* < 0.001, d = 0.60) and trial 4 (*p* < 0.001, d = 0.83), and lastly, trial 3 was different than trial 4 (*p* < 0.001, d = 0.46).

Bonferroni-corrected post-hoc tests indicated that for the between-subject factor, reminder with exercise was not different than reminder (*p* = 0.99, d = 0.06), no reminder (*p* = 0.99, d = 0.10) or interference control (*p* = 0.17, d = 0.25). Reminder was not different than no reminder (*p* = 0.99, d = 0.04) but was different than interference control (*p* = 0.03, d = 0.31). Similarly, no reminder was different than interference control (*p* = 0.01, d = 0.36).

#### 3.1.6. Intrusions from List 2

In a 3 (groups) × 4 (learning trials) mixed-ANOVA, with the group as the between-subjects variable and the learning trials (1–4) as the within-subject variable, there were no main effect for group, *F*(2, 57) = 0.13, *p* = 0.87, η^2^_p_ = 0.005, main effect for the learning trials, *F*(2.18, 124.23) = 0.07, *p* = 0.94, η^2^_p_ = 0.001, and no group by learning trials interaction, *F*(4.35, 124.23) = 2.02, *p* = 0.08, η^2^_p_ = 0.06.

## 4. Discussion

Although emerging work suggests that acute exercise can enhance episodic memory function, limited research has examined whether acute exercise can attenuate memory distortion from memory reactivation. As such, in this experiment, memory reactivation was induced, followed by a high-intensity bout of acute exercise. We hypothesized that after memory reactivation, engaging in an acute bout of high-intensity exercise would result in greater delayed memory performance when compared to memory reactivation without exercise. Our results partially support this hypothesis. That is, the reminder with exercise group recalled more words on Visit 3 than the reminder group, but these means were not statistically significantly different from each other, and as such, should be interpreted accordingly. However, on Visit 3, the interference control group recalled significantly more words than the no reminder group and the reminder group, but there was no statistical difference between the interference control and reminder with exercise group.

Limited research has examined the effects of acute exercise on memory reconsolidation. In a rodent model, a single bout of high-intensity exercise was shown to enhance acquisition, extinction, and reconsolidation of context conditioned fear [29]. To our knowledge, only one human study has evaluated this paradigm [30]. Keyan and Bryant [30] conducted a 3-day reconsolidation paradigm and showed that a 20–25 min bout of incremental cycling after memory reactivation was associated with greater central detail recall from a trauma film depicting the aftermath of a highway car crash. However, acute exercise did not enhance peripheral detail recall or intrusive memory. In our experiment, the reminder with exercise group was not significantly different than the interference control group, which suggests that exercise may, potentially, attenuate memory distortion effects from memory reactivation. However, this assertion needs to be interpreted cautiously, as delayed memory in the reminder with exercise group was not different than that in the reminder and no reminder groups.

Given the paucity of research on this paradigm, future work is needed. The memory type of our experiment differed from that of Keyan and Bryant. That is, we evaluated non-emotional memory, whereas they focused on emotional memory. Research demonstrates that acute exercise is associated with both non-emotional [31,32] and emotional memory [33]. As such, it is plausible that acute exercise may enhance memory reconsolidation for both non-emotional and emotional memories. We specifically chose not to employ an emotional memory paradigm with the current experiment, as our recent work has not been effective in enhancing emotional memory from acute exercise [34]. It would also be of interest for future work to consider varying the timing of the acute bout of exercise after memory reactivation. Perhaps, at least for non-emotional memory, engaging in acute exercise immediately after memory reactivation is not the optimal time to facilitate reconsolidation. In a recent experiment employing a non-emotional paradigm, we showed that exercising 4 h after memory encoding was effective in facilitating long-term memory function [15]. In support of this, in a recent meta-analysis [1], we observed a greater effect size for enhancing long-term memory if the acute bout of exercise occurred later in the consolidation period.

In conclusion, the present study extends this under-investigated paradigm by evaluating whether acute exercise, after memory reactivation, can enhance delayed intentional memory. We did not observe strong evidence of such an effect. Future work is needed to confirm our findings, as well as extend this line of inquiry by evaluating different memory types, consider different exercise intensities and temporal periods of acute exercise after memory reactivation, and evaluate if this paradigm is moderated by factors such as biological sex [35] and BDNF (brain-derived neurotrophic factor) polymorphism [13,14,36]. Such mechanistic work is needed, as several past studies do not demonstrate a mediational effect of, for example, BDNF, on the exercise–cognition interaction [37,38,39].

## Figures and Tables

**Table 1:** Lists of objects presented on Visit 1 and Visit 2.

List 1	List 2
Block	Band-Aid
Bow	Battery
Car (toy)	Book
Coin (quarter)	Box
Crayon	Brush
Cup	Diaper
Dice	Doll
Dinosaur	Dollar Bill
Flashlight	Envelope
Glue	Glove
Knife	Hanger
Life Saver Candy	Rock
Microphone	Staples
Pencil	Straw
Ponytail Holder	Tissue
Sock	Thread
Sunglasses	Tomato
Teabag	Toy Pot
Tennis Ball	Tupperware Lid
Zip Lock Bag	Watch

**Table 2:** Study protocol overview.

Start	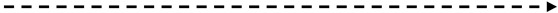					Finish
GROUP	Visit 1		Visit 2			Visit 3
Reminder w/Exercise	List 1 Memory Encoding and Retrieval (to criterion)	48 h break	Reminded of List 1 protocol (no retrieval) and then encoded and retrieved List 2 to criterion	15 min high-intensity exercise	48 h break	List 1 retrieval (4 trials)
Reminder	List 1 Memory Encoding and Retrieval (to criterion)	48 h break	Reminded of List 1 protocol (no retrieval) and then encoded and retrieved List 2 to criterion	No exercise	48 h break	List 1 retrieval (4 trials)
No Reminder	List 1 Memory Encoding and Retrieval (to criterion)	48 h break	No reminding of List 1 protocol; different researcher for this visit and in a different lab. Encoded and retrieved List 2 to criterion	No exercise	48 h break	List 1 retrieval (4 trials)
Interference Control	List 1 Memory Encoding and Retrieval (to criterion)	96 h break				List 1 retrieval (4 trials)

* During all between-day break periods, participants were instructed not to engage in high-intensity exercise.

**Table 3:** Demographic characteristics of the sample.

Variable	Reminder with Exercise	Reminder	No Reminder	Interference Control	*p*-Value
*N*	20	20	20	20	
Age, mean years	20.10 (0.9)	20.58 (1.7)	21.10 (4.2)	20.30 (1.4)	0.61
Gender, % female	50.0	78.9	76.2	65.0	0.20
Race–Ethnicity, % white	90.0	89.5	81.0	95.0	0.57
BMI, mean kg/m^2^	24.02 (2.4)	24.62 (4.9)	26.30 (5.0)	24.82 (3.6)	0.35

BMI, Body mass index.
